# Study of High-Temperature Behaviour of ZnO by Ab Initio Molecular Dynamics Simulations and X-ray Absorption Spectroscopy

**DOI:** 10.3390/ma14185206

**Published:** 2021-09-10

**Authors:** Dmitry Bocharov, Inga Pudza, Konstantin Klementiev, Matthias Krack, Alexei Kuzmin

**Affiliations:** 1 Institute of Solid State Physics, University of Latvia, Kengaraga Street 8, LV-1063 Riga, Latvia; inga.pudza@cfi.lu.lv; 2 MAX IV Laboratory, Lund University, P.O. Box 118, SE-221 00 Lund, Sweden; konstantin.klementiev@maxiv.lu.se; 3 Laboratory for Materials Simulations, Paul Scherrer Institut (PSI), CH-5232 Villigen, Switzerland; matthias.krack@psi.ch

**Keywords:** ZnO, zinc oxide, ab initio molecular dynamics, extended X-ray absorption fine structure

## Abstract

Wurtzite-type zinc oxide (w-ZnO) is a widely used material with a pronounced structural anisotropy along the *c* axis, which affects its lattice dynamics and represents a difficulty for its accurate description using classical models of interatomic interactions. In this study, ab initio molecular dynamics (AIMD) was employed to simulate a bulk w-ZnO phase in the NpT ensemble in the high-temperature range from 300 K to 1200 K. The results of the simulations were validated by comparison with the experimental Zn K-edge extended X-ray absorption fine structure (EXAFS) spectra and known diffraction data. AIMD NpT simulations reproduced well the thermal expansion of the lattice, and the pronounced anharmonicity of Zn–O bonding was observed above 600 K. The values of mean-square relative displacements and mean-square displacements for Zn–O and Zn–Zn atom pairs were obtained as a function of interatomic distance and temperature. They were used to calculate the characteristic Einstein temperatures. The temperature dependences of the O–Zn–O and Zn–O–Zn bond angle distributions were also determined.

## 1. Introduction

Zinc oxide (ZnO) is a wide band-gap ($E_{g}$ = 3.37 eV) semiconductor, which has a wide range of technological applications, making it an extremely popular research topic in recent years [1,2,3,4,5,6,7]. The crystal lattice of the most common ZnO phase belongs to the wurtzite type and is strongly anisotropic [8,9,10], giving origin to its piezoelectric and pyroelectric properties [11,12,13,14,15,16].

The unit cell of the wurtzite-type ZnO (w-ZnO) is hexagonal with the space group $P6_{3}mc$ (186). Each type of atom (Zn or O) forms a hexagonal close-packed sublattice displaced relative to each other along the third-order *c* axis by the parameter *u* [1]. The Wyckoff positions (2*b*) of the Zn and O atoms in the unit cell are Zn (0, 0, 0), (1/3, 2/3, 1/2) and O (0, 0, *u*), (1/3, 2/3, 1/2+*u*). The coordination of each atom in w-ZnO is tetrahedral by four atoms of the other type, and the parameter *u* determines the distortion of the ZnO${}_{4}$ tetrahedra. The anisotropic structure of ZnO affects its lattice dynamics. In particular, the anisotropy of ZnO thermal expansion and atom thermal vibrations (thermal ellipsoids) along the *a* and *c* axes directions was observed by X-ray diffraction [17,18,19].

Phonons control all thermal properties of ZnO such as heat capacity, thermal expansion and thermal conductivity [20]. The latter is a key factor responsible for heat dissipation and thus limits the use of ZnO in power electronics applications [21]. Therefore, an understanding of the phonon dynamics in the high-temperature regime is important for the design of ZnO-based devices.

The lattice dynamics of wurtzite-type ZnO was studied in the past by the Zn K-edge X-ray absorption spectroscopy at low temperatures (10–300 K) [22,23]. The information on the thermal disorder and anisotropy effects was extracted using two different simulation approaches, such as classical molecular dynamics and reverse Monte Carlo (RMC), which were both combined with ab initio multiple-scattering (MS) theory. The accuracy of several force-field models [24,25,26] often used for molecular dynamics simulations of bulk and nanocrystalline ZnO was confirmed by comparing the experimental and simulated Zn K-edge extended X-ray absorption fine structure (EXAFS) spectra. It was found that the existing force-field models cannot accurately describe the correlated atomic motion. At the same time, a more accurate solution was obtained from EXAFS data with the RMC method. In particular, two non-equivalent groups of atoms were resolved in both the first and second coordination shells of the absorbing Zn atom as a result of the ZnO structure anisotropy. An increase in temperature leads to the fact that the structure of ZnO becomes even more anisotropic, which is reflected in a change in the parameter *u* [22]. As a result, oxygen atoms displace along the *c* axis, and the Zn–O bond lengths vary [22].

Ab initio molecular dynamics (AIMD) provides an alternative, although computationally more expensive, approach to describe the lattice dynamics in zinc oxide. In this study, we performed AIMD simulations for bulk wurtzite-type ZnO at high temperatures (300–1200 K) and validated the obtained theoretical results by their direct comparison with the experimental Zn K-edge EXAFS spectra.

## 2. Experimental Details

Polycrystalline wurtzite-type ZnO (99.99% purity, Alfa Aesar, Haverhill, MA, USA) and BN powders were mixed and pressed into a pellet. High-temperature Zn K-edge (9659 eV) X-ray absorption experiments were performed on the BALDER beamline at the 3.0 GeV storage ring of MAX IV Laboratory [27]. The X-ray beam from the in-vacuum wiggler source was monochromatized using a liquid-nitrogen-cooled double-crystal Si(111) monochromator and measured by two ionization chambers located before and after the sample and filled with N${}_{2}$ and Ar gases. An uncoated Si collimating mirror and silica focusing mirror were used for harmonic reduction. The sample temperature was controlled using the Linkam, 1000 ${}^{\circ }$C furnace.

## 3. Computational Details

The AIMD simulations of wurtzite-type ZnO were performed within the isobaric-isothermal (NpT) ensemble. The trajectories of atoms were obtained by numerically solving Newton’s equations of motion, and the forces between atoms were calculated on-the-fly based on Kohn–Sham density functional theory (DFT) within the Born–Oppenheimer approximation. All simulations were conducted using the Quickstep module [28] of the CP2K code [29,30] at the Swiss National Supercomputing Centre (CSCS, “Piz Daint”). The AIMD simulations were performed at four temperatures: 300 K, 600 K, 900 K and 1200 K. At each temperature, the system was first thermalized for 15 ps, and the atomic configurations were collected during the production (sampling) run of 30 ps duration. The exchange-correlation functional PBEsol [31] was employed in all runs together with Goedecker–Teter–Hutter (GTH) pseudopotentials ([Ar] $4s^{2}\;3d^{10}$ for Zn and [He] $2s^{2}\;2p^{4}$ for O) [32] and contracted Gaussian basis sets of double-zeta quality. The basis sets were specifically optimized for use with the GTH pseudopotentials for the condensed phase systems (DZVP, MOLOPT-SR) [33].

**Figure 1:**
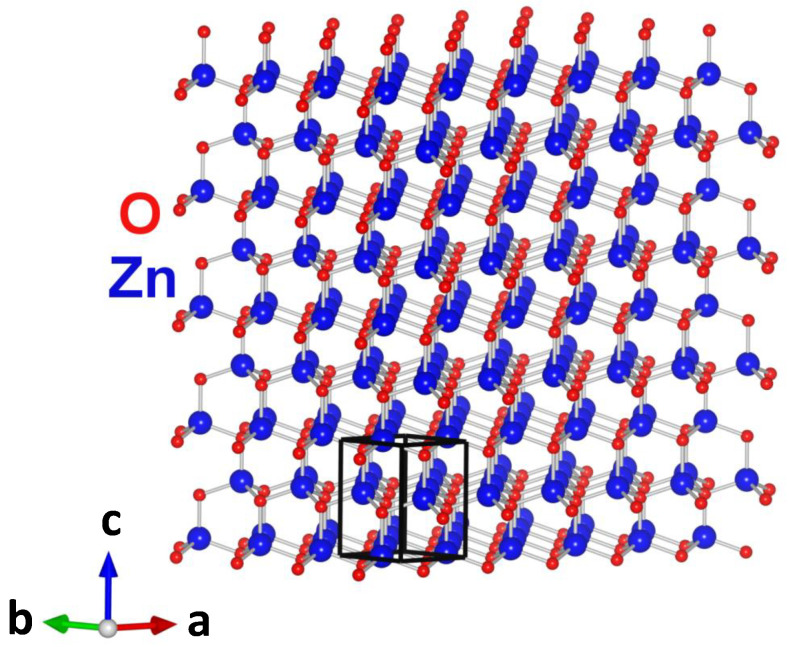
The crystal structure of wurtzite-type ZnO: a supercell 6*a* × 4*b* × 4*c* used in AIMD simulations is shown. The unit cell is indicated by thick black lines.

The zinc oxide structure was modelled by a 6a×4b×4c (a=b=3.2417 Å, c=5.1876 Å [19]) supercell, composed of 96 orthorhombic unit cells (Figure 1). Periodic boundary conditions (PBC) were employed in all runs. Note that to avoid artificial correlation effects due to PBC in EXAFS calculations, the maximum cluster radius was set to half of the shortest supercell vector length [34,35]. An orthorhombic unit cell was used because the CP2K/Quickstep implementation works computationally more efficiently for such supercells that speed up the AIMD simulations. A DFT+U correction [36,37,38] was applied for all zinc atoms using an effective Hubbard $U_{\mathrm{eff}}\left(\mathrm{Zn}\right)$ of 1.0 eV.

An ensemble of uncorrelated atomic configurations retrieved from the AIMD trajectory at each temperature was used to calculate the configuration-averaged Zn K-edge EXAFS χ(k). Here *k* is the photoelectron wavenumber defined as $k=\sqrt{\left(2m_{e}/\hbar^{2}\right)\left(E-E_{0}\right)}$, where $m_{e}$ is the electron mass, and *ℏ* is the Planck constant. The origin $E_{0}$ of the kinetic energy of the photoelectron was set to obtain the best alignment between the energy scales of the experimental and calculated EXAFS spectra [39]. All EXAFS calculations were performed within the multiple-scattering formalism [34,40] using the ab initio self-consistent real-space MS FEFF8.50L code [41,42]. The cluster with the radius of 8 Å and centred at the absorbing Zn atom was constructed based on the crystallographic ZnO structure and used to obtain the potential and partial phase shifts, required for the calculation of scattering amplitudes in the EXAFS equation [39]. The EXAFS amplitude damping due to the photoelectron inelastic losses was accounted for using the complex exchange-correlation Hedin–Lundqvist potential [43]. The EXAFS amplitude reduction factor $S_{0}^{2}$ was set to 1.0 [39].

## 4. Results and Discussion

The average lattice parameters obtained using the AIMD calculations in the NpT ensemble increase with temperature and are in agreement with the known experimental values (Table 1). Thus, AIMD simulations reproduce well the thermal expansion of the lattice. Note that there is some scattering in the experimental values of the lattice parameters of ZnO at 300 K [44].

**Figure 2:**
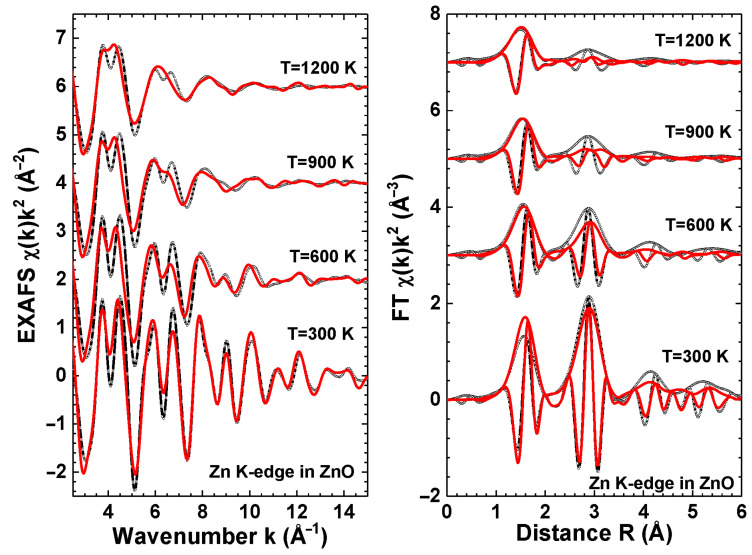
Comparison of the experimental (black curves) and AIMD calculated (red curves) Zn K-edge EXAFS spectra and their Fourier transforms (FTs) at 300, 600, 900 and 1200 K. The AIMD calculations were performed in the NpT ensemble. The curves are shifted vertically for clarity.

The Zn K-edge EXAFS $\chi \left(k\right)k^{2}$ spectra and their Fourier transforms (FTs) calculated at 300, 600, 900 and 1200 K based on the results of the AIMD simulations using the NpT ensemble are shown in Figure 2 in comparison with the experimental data. Note that the peaks in the FTs are located at the distances *R* which are slightly shorter than crystallographic distances *r* because the FTs were not corrected for the phase shift present in the EXAFS equation [39]. On the whole, the calculated EXAFS spectra $\chi \left(k\right)k^{2}$ reasonably reproduce the experimental ones at all temperatures. The calculated and experimental EXAFS spectra agree well in phase in *k*-space as well as in the position of peaks in their FTs in *R*-space due to the small difference between the lattice parameters, which remains less than 0.02 Å at all temperatures (Table 1).

An increase in the damping of the EXAFS spectra at large-*k* values is clearly seen in Figure 2 at higher temperatures and is due to stronger thermal disorder. The comparison of the results in *R*-space allows for an estimation of the disorder effects in the different coordination shells. As one can see, the experimental and calculated FTs agree well in the first coordination shell of zinc composed of four oxygen atoms (the peak at *R* = 1.6 Å, uncorrected), however, the thermal disorder is slightly overestimated by AIMD in the outer coordination shells leading to the smaller peak amplitude at large *R*-values. Some disagreement in the first peak amplitude for *T* = 300 K can be caused by the remaining contribution of quantum effects (zero-point energy), which is not taken into account in our AIMD simulations and becoming negligible at higher temperatures.

**Figure 3:**
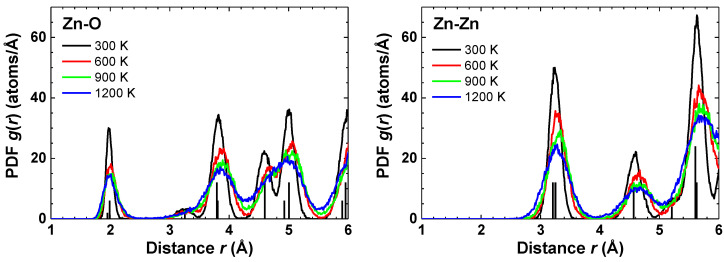
Atomic pair distribution functions (PDFs) around absorbing Zn atom obtained from AIMD calculations in the NpT ensemble at 300, 600, 900 and 1200 K. Vertical lines show crystallographic distances in wurtzite-type ZnO at 300 K from [19].

A comparison of the pair distribution functions (PDFs) g(r) for Zn–O and Zn–Zn atom pairs, calculated from the atomic coordinates, are shown in Figure 3. The crystallographic distances at 300 K are indicated by vertical lines [19]. The first coordination shell of zinc is strongly asymmetric at 600, 900 and 1200 K, indicating the anharmonicity of the Zn–O bonding. This result agrees well with the experimental findings by single-crystal neutron diffraction [19].

**Figure 4:**
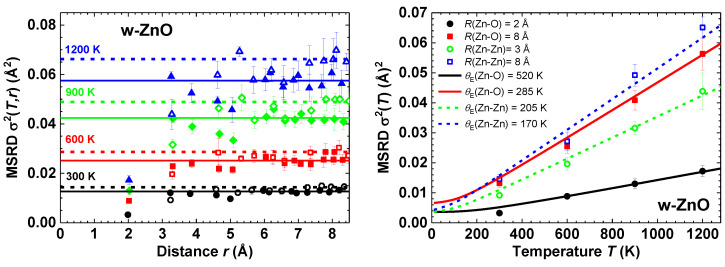
Left panel: Dependence of the Zn–O (solid symbols) and Zn–Zn (open symbols) MSRDs $\sigma {\left(T,r\right)}^{2}$ on distance *R* and temperature *T* in wurtzite-type ZnO evaluated from AIMD calculations at 300 K (circles), 600 K (squares), 900 K (diamonds) and 1200 K (triangles) in the NpT ensemble. Horizontal lines show asymptotic behaviour of MSRDs for distant shells. Right panel: Temperature dependence of MSRD factors for the nearest and distant Zn–O and Zn–Zn atom pairs in w-ZnO. Solid and dashed lines are fits by the Einstein model with the characteristic temperature $\theta_{E}$ for Zn–O and Zn–Zn atom pairs, correspondingly.

The atomic coordinates from the AIMD simulations were used to calculate the mean-square relative displacements (MSRDs) $\sigma^{2}$ for each pair of atoms and to follow their distance and temperature dependencies. The values of $\sigma^{2}\left(T,r\right)$ for Zn–O and Zn–Zn atom pairs are shown in Figure 4 (left panel). Note that MSRD values increase with temperature and interatomic distance.

It is known that the MSRD value for the pair of atoms *i* and *j* is related to their mean-square displacements (MSDs) as ${\mathrm{MSRD}}_{ij}={\mathrm{MSD}}_{i}+{\mathrm{MSD}}_{j}-2\varphi \sqrt{{\mathrm{MSD}}_{i}}\sqrt{{\mathrm{MSD}}_{j}}$, where φ is a dimensionless correlation parameter [45,46]. Therefore, the behaviour of MSRDs at large distances (r≳ 5–6 Å) in Figure 4 reflects the disappearance of correlation ($\varphi \overset{r⟶\infty }{\to }0$) in the motion of distant atoms [46,47,48,49]. Therefore, the asymptotic behaviour of MSRD can be utilized to determine MSD values (Table 2). The MSD values at 300, 600 and 900 K are in agreement with the experimental ones obtained by single-crystal neutron diffraction [19].

The temperature dependencies of the MSRDs for the nearest and distant Zn–O and Zn–Zn atom pairs were fitted using the Einstein model [50] (Figure 4 (right panel)). The obtained values of the characteristic Einstein temperatures $\theta_{E}$ are also reported. Large values of $\theta_{E}$ for the nearest O and Zn atoms reflect stronger interactions in the atomic pairs and correlated motion of atoms. At large distances, the interaction between atoms weakens, and their motion becomes uncorrelated, which is reflected in a significant decrease of $\theta_{E}$. The difference between the two $\theta_{E}$ values is significantly larger for Zn–O compared to Zn–Zn interactions which is a result of the chemical bonding between the Zn and O atoms.

**Figure 5:**
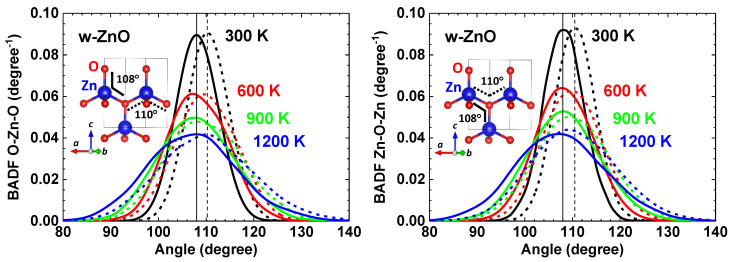
Bond angle distribution functions (BADFs) O–Zn–O and Zn–O–Zn within and between ZnO${}_{4}$ tetrahedra, respectively, calculated by AIMD. Dashed (solid) curves correspond to the O–Zn–O and Zn–O–Zn angles within the ab plane (along the *c* axis). Vertical lines indicate the positions of the distribution maxima at 300 K.

The local lattice dynamics in wurtzite-type ZnO can also be described by a variation of the O–Zn–O and Zn–O–Zn bond angles within and between ZnO${}_{4}$ tetrahedra, respectively (Figure 5). One can distinguish two different O–Zn–O (Zn–O–Zn) angles related to the atomic chains located in the ab plane or along the *c* axis. The calculated mean values of the two angles at 300 K are about 110${}^{\circ }$ in the ab plane and about 108${}^{\circ }$ along the *c* axis, indicating that the Zn atoms are displaced closer to three oxygen atoms located in the basal plane. An increase of temperature leads to the broadening of the bond angle distributions and to a slight decrease of their mean values by about 1${}^{\circ }$ at 1200 K. The obtained values of bond angles at 300, 600 and 900 K are in good agreement with the experimental ones obtained by single-crystal neutron diffraction [19].

## 5. Conclusions

AIMD simulations of wurtzite-type ZnO were performed in the NpT ensemble at 300, 600, 900 and 1200 K. The simulation results were validated by comparison with the experimental Zn K-edge EXAFS spectra. Upon increasing temperature, a unit cell expansion occurs in agreement with the available experimental data [19] (Table 1). The obtained atomic configurations were used to calculate the configuration-averaged EXAFS spectra within the multiple-scattering approach [22,23] and are in qualitative agreement with the experimental ones.

Based on the results of the AIMD simulations, the mean-square relative displacements (MSRDs) for Zn–O and Zn–Zn atom pairs and the mean-square displacements (MSDs) for Zn and O atoms were determined as a function of interatomic distance and temperature. The obtained values of MSDs agree well with those found by single-crystal neutron diffraction [19]. The characteristic Einstein temperatures were determined for Zn–O and Zn–Zn atom pairs from the temperature dependencies of MSRDs.

The PDFs for Zn–O and Zn–Zn show a significant temperature dependence due to the lattice expansion and the amplitude change of the atomic vibrations (Figure 3). An increase of temperature leads to the broadening of the bond angle distributions (Figure 5), whereas the mean values of the bond angles O–Zn–O and Zn–O–Zn remain almost temperature independent. This fact suggests a weak temperature dependence of the parameter *u*(O) and the piezoelectric coefficients [8]. Our results are in agreement with the conclusions in [19] based on the neutron diffraction measurements in the temperature range of 20–900 K.

## Figures and Tables

**Table 1:** The average lattice parameters for ZnO obtained from AIMD simulations using the NpT ensemble vs. the experimental values from [19].

	AIMD		Experiment	
Temperature (K)	*a* (Å)	*c* (Å)	*a* (Å)	*c* (Å)
300	3.258	5.220	3.24992 (5)	5.20658 (8)
600	3.265	5.231	3.25682 (5)	5.21251 (8)
900	3.272	5.243	3.26480 (5)	5.21939 (8)
1200	3.281	5.257		

**Table 2:** Temperature dependence of the mean-square displacements (MSDs) for oxygen and zinc atoms in ZnO, estimated from the asymptotic behaviour of MSRDs at large distances.

T (K)	MSD (O) (Å${}^{2}$)	MSD (Zn) (Å${}^{2}$)
300	0.0054 (4)	0.0072 (4)
600	0.010 (3)	0.014 (3)
900	0.018 (5)	0.024 (5)
1200	0.024 (6)	0.033 (6)

## Data Availability

The data presented in this study are available in article.

## Abbreviations

The following abbreviations are used in this manuscript:

AIMD	Ab Initio Molecular Dynamics
BADF	Bond Angle Distribution Functions
DFT	Density Functional Theory
EXAFS	Extended X-ray Absorption Fine Structure
FT	Fourier Transform
MS	Multiple-Scattering
MSD	Mean-Square Displacements
MSRD	Mean-Square Relative Displacements
PDF	Pair Distribution Function
RMC	Reverse Monte Carlo
